# Supplementary material for: Predictors of elevated carotid intima-media thickness as a surrogate marker for cardiovascular disease in pediatric chronic kidney disease
Source: PLoS One. 2025 Aug 8;20(8):e0329412. doi: 10.1371/journal.pone.0329412 (PMC12333978; doi:10.1371/journal.pone.0329412)
Supplement: Attachment — Submitted filename: [PLOS one] Response to the reviewers.docx [file pone.0329412.s002.docx]

PONE-D-24-25206

**Predictors of increased vascular thickness in children with chronic kidney disease**

Response to the reviewers

**Reviewer 1**

**Comments to the Author**

Thank you very much for inviting me as a peer reviewer and giving me the opportunity to review. The author studied the predictive factors of increased vascular thickness in children with chronic kidney disease. This research is of great significance for reducing cardiovascular events in children with CKD in the future. The content of the article still needs further modification to arouse more interest from readers.

**Introduction?**

**There is a lack of connection between the first paragraph and the second paragraph. The first paragraph of this article tells the reason for the high mortality rate of CKD. The second paragraph directly leads to the acceleration of atherosclerosis and cardiovascular disease. The lack of connection of relevant content may confuse readers. Suggest the author to add reasons (such as high CKD mortality rate, which may be caused by factors such as XX, XX, etc., with cardiovascular factors being the main factor).**

**Authors’ response**

Thank you for the suggestion to revise the wording and flow of the manuscript. We have amended the sentences as suggested.

**Change made in the manuscript**

*The elevated mortality risk is largely attributed to factors such as accelerated atherosclerosis, vascular calcification, and chronic inflammation, which are common in CKD patients. Among these, cardiovascular disease stands out as a major in individuals with end-stage CKD.* (Page 3, line 45 – 47)

**Methods?**

1. **The sample size of this study is 71 cases. How did the author determine the sample size? It is not provided in the manuscript.**
2. **Was blinding used in this study? If used, is it single blind or double-blind? Please explain to the author.**
3. **What is the deadline for measuring CIMT at least three months after CKD diagnosis? Is follow-up conducted after CIMT and echocardiography measurements? If so, how long is the follow-up period?**

**Authors’ response**

Thank you for the suggestion to improve the method section. We have modified the related points as suggested.

1. Sample size calculation have been added in the method section.
2. This study is a single blind study who is the trained technicians conducting the echocardiography assessment, we added this information within the manuscript.
3. No follow up period after CIMT measurement was conducted within this study, this limitation was listed within the discussion.

**Change made in the manuscript**

1. *The sample size calculation was based on a 74.5% prevalence of elevated CIMT in children with CKD, considering five independent variables. Following the rule of thumb for factor analysis (at least 10 events per predictor), a minimum of 67 patients was required* (Page 4, line 78 – 80)
2. *As a part of single-blind study design, the measurement was done by trained technicians who were blinded to the participant’s clinical information.* (Page 6, line 120-121)
3. *None*

**Results?**

**Could the author explain the main outcome measure of this study? Is it CIMT? Please explain and make corresponding modifications to the abstract section.**

**Authors’ response**

This study aimed to identify predictors of cardiovascular events in pediatric patients with CKD, using CIMT as a surrogate marker. The independent variable was the predictors of elevated CIMT, while the dependent variable (outcome) was CIMT. This outcome has been incorporated into the abstract (last sentence of the background), as well as the introduction and methods sections.

**Change made in the manuscript**

*Abstract:*

*Carotid intima-media thickness (CIMT) was the outcome measure, assessed at least three months post-diagnosis to evaluate cardiovascular events based on vascular thickness* (Page 2, line 24-26)*.*

*Method section:*

*The independent variable was the predictors of elevated CIMT, while the dependent variable (outcome) was CIMT* (Page 7, line 144-145).

**Discussion?**

**What is the possible reason for the statistical difference in the systolic and diastolic function of the heart between the CIMT elevated group and the non elevated group? Please add relevant literature to enhance the persuasiveness of the content.**

**Authors’ response**

As provided in the Result section, page 10 line 169-170, this study revealed that systolic and diastolic functions showed no statistical differences between groups with and without elevated CIMT.

**Change made in the manuscript**

*None*

**Other parts?**

**Is the relevant data in this article shared? Please indicate at the end of the article that the author.**

**Authors’ response**

Data used in this study is available upon request.

**Change made in the manuscript**

*Data used in this study is available upon request.* (Page 8, line 154)

**Reviewer 2**

**Comments to the Author**

Thank you for inviting me to review this manuscript. Overall, the study gets 2 out of 5 points regarding its novelty. I think the manuscript needs a major revision and cannot be considered for publication without addressing the following points:

1. **The title of the study does not accurately reflect its content and should be revised for better alignment.**

**Authors’ response**

Thank you for the feedback, we have modified the title and replaced it with “Predictors of elevated carotid intima-media thickness as a surrogate marker for cardiovascular disease in pediatric chronic kidney disease”

**Change made in the manuscript**

*Predictors of elevated carotid intima-media thickness as a surrogate marker for cardiovascular disease in pediatric chronic kidney disease* (Page 1, Line 1-2)

2. **Remove the sentences saying that this study is the first study on this topic.**

**Authors’ response**

Thank you for the suggestion to remove the specific sentence, we have modified it as suggested.

**Change made in the manuscript**

*As far as we are aware, limited studies have explored predictive factors for cardiovascular events in children with CKD in Indonesia* (Page 4, Line 66-67).

3. **The authors describe their study as a retrospective cohort; however, the duration of follow-up is unclear. If no follow-up was conducted, please clarify how the relative risk (RR) was calculated.**

**Authors’ response**

Our retrospective cohort study used multivariate logistic regression to identify independent predictors at diagnosis (e.g., age, male sex, obesity, CKD stage, and hypertension) associated with elevated CIMT (outcome), measured at least three months post-diagnosis as a proxy for cardiovascular risk. Adjusted odds ratios (OR) were obtained from the analysis, as stated in the abstract. We have also corrected the RR in Table 3 as suggested.

**Change made in the manuscript**

***Abstract****: We used multivariate logistic regression to identify independent predictors at diagnosis (e.g., age, male sex, obesity, CKD stage, and hypertension) associated with elevated CIMT (outcome), measured at least three months post-diagnosis as a proxy for cardiovascular risk* (Page 2, line 24-26).

**Table 3:** *OR: Odd Ratio* (Page 11, Line 182-183).

4. **Line 46: The time period for which this mortality rate applies is missing. Please specify the year.**

**Authors’ response**

Thank you for the kind correction, we have added the time period of the mortality rate in the manuscript.

**Change made in the manuscript**

*In 2018, mortality rates for children aged 1-19 years with end-stage CKD in general US pediatric population were reported at 0.31 per 1000 population.*  (Page 3, Line 49-50)

5. **Methods: It is recommended to include details about the ultrasound device used in the study.**

**Authors’ response**

Thank you for the recommendation, we have added the details of the ultrasound device in the method section.

**Change made in the manuscript**

*The CIMT measurements were conducted using a common carotid artery B-mode ultrasound with a Phillips HD11 XE ultrasound system, a fully digital imaging platform designed for high-definition vascular imaging. It was equipped with an L12-3 broadband linear array transducer, which operates at frequencies of 3–12 MHz and features a 38 mm aperture for precise assessments of vascular structures. This combination enables detailed imaging and ensures accurate evaluation of CIMT in pediatric patients [14].*  (Page 6-7, Line 125-131)

6. **Methods: A reference for the assessment of nutritional status should be cited.**

**Authors’ response**

We have added the citation for nutritional status assessment in the method section as advised.

**Change made in the manuscript**

*Nutritional status was evaluated using height and weight measurements, applying the WHO Growth Reference 2007 definitions through WHO AnthroPlus software [11]* (Page 5, Line 91-92).

7. **Methods: Please provide details on how height and weight measurements were conducted.**

**Authors’ response**

Thank you for the advise, we have added the details of related measurements as suggested.

**Change made in the manuscript**

*Height measurement in children who can stand were measured using stadiometer with the child stands barefoot with heels, buttocks, shoulders, and head touching the vertical board. For children who were not able to stand, recumbent length is measured using an infantometer, with the child lying flat, and the head and feet aligned to the measurement board. While for weight measurement, a calibrated digital scale were used. Measurements were taken with minimal clothing and no shoes to avoid interference with the reading. The nutritional status classifications used were…*  (Page 5, Line 92-98).

8. **The limitations section should be expanded, as the study has additional limitations that were not fully addressed.**

**Authors’ response**

Thank you for your review, we have expanded the limitations in the discussion section.

**Change made in the manuscript**

*Furthermore, the study did not account for other predictors such as dietary habits, physical activity levels, or genetic predisposition, which could influence vascular thickness independently of CKD. These limitations suggest the need for cautious interpretation of the findings regarding generalizability and longitudinal CIMT trends, and the role of external risk factors in pediatric CKD patients.*  (Page 13, Line 225-229)
