# Supplementary material for: Predictors of elevated carotid intima-media thickness as a surrogate marker for cardiovascular disease in pediatric chronic kidney disease
Source: PLoS One. 2025 Aug 8;20(8):e0329412. doi: 10.1371/journal.pone.0329412 (PMC12333978; doi:10.1371/journal.pone.0329412)
Supplement: Attachment — Submitted filename: [PLOS_one]_Response_to_the_reviewers_auresp_2.docx [file pone.0329412.s003.docx]

PONE-D-24-25206R1

**Predictors of elevated carotid intima-media thickness as a surrogate marker for cardiovascular disease in pediatric chronic kidney disease**

Response to the reviewers

**Reviewer #1**

**Comments to the Author**
Thank you to the author for responding carefully to the first round of review comments and completing the revisions. The article has made significant improvements in methodological description, data presentation, and discussion depth, but there are still the following issues that need further improvement:

**Discussion section of the article:**

1. Need to respond to the latest evidence-based medicine evidence: contradictory conclusions regarding the predictive value of CIMT compared to CKD studies published in 2023

**Authors’ response**

Thank you for the kind suggestion to respond to the latest evidence-based medicine evidence. We have added and cited the article as suggested into the discussion section.

**Change made in the manuscript**

*A 2023 case-control study involving children with CKD stages 3-4 reported contradictory findings regarding the predictive value of CIMT. The study found no significant differences in CIMT measurements between CKD cases and healthy controls [21]. In contrast, a study in adults with advanced CKD, CIMT was significantly associated with lower estimated glomerular filtration rate (eGFR) and increased cardiovascular risk, supporting its role as a predictive marker [22]. However, in pediatric CKD patients, CIMT showed no significant correlation with CKD stage or cardiovascular risk factors [23]. Meanwhile, findings in predialysis CKD were mixed—some studies supported CIMT as a predictor of cardiovascular events, while others found carotid plaque burden to be more reliable. These inconsistencies highlight the need for population-specific approaches and further research to clarify CIMT’s value in CKD management [24].* (Page 13, line 212 – 221)

**Discussion section of the article:**

2. Clinical translation suggestion: Provide specific pathways for integrating CIMT testing into the existing cardiovascular risk assessment system for CKD children, such as screening frequency and threshold definition.

**Authors’ response**

Thank you for the suggestion to enrich the discussion in this article. As recommended, we have added to the manuscript a specific pathway that integrates CIMT examination into the current system for assessing cardiovascular in children with CKD.

**Change made in the manuscript**

*To support practical clinical integration, CIMT assessment should become part of routine cardiovascular risk evaluation in children with CKD. Annual CIMT screening is recommended for those with CKD stages 3-5, and every two years for stages 1-2. CIMT values at or above the 95^th^ percentile (adjusted for age, sex, and height) are considered abnormal and signal increased cardiovascular risk. An elevated CIMT should trigger further evaluation, including blood pressure monitoring and echocardiographic assessment for cardiac structure and function. Children with both elevated CIMT and other risk factors, such as hypertension, should be prioritized for intensified interventions, including tighter blood pressure control, lifestyle changes, and targeted pharmacologic therapy. This integrated approach supports early identification and management of high-risk patients, aiming to improve long-term cardiovascular outcomes in pediatric CKD.* (Page 13, line 223 – 232)

**Discussion section of the article:**

3. Given the racial differences in the normal reference values of CIMT in children, it is recommended to supplement the racial composition of the study population in the limitations section.

**Authors’ response**

Thank you for highlighting the importance of considering racial differences in measuring CIMT in children. As suggested, we have included this issue on the limitations section.

**Change made in the manuscript**

*Our cohort compromised predominantly Southeast Asian children, which may limit generalizability to populations with differing CIMT reference norms. Future multiracial studies might be needed to validate a more universal CIMT thresholds.* (Page 14, line 246 – 249)

**Reference section:**

Due to some of the references being relatively old, it is recommended that the author update 3-5 relevant references from the past 3 years.

**Authors’ response**

Thank you for the suggestion. We have added updated relevant references from the past three years to statements previously supported by relatively older articles. Additional new references have also been included to support Discussion section, as outlined in response #1 to reviewer.

**Change made in the manuscript
*Within Introduction section:***

*Chronic kidney disease (CKD), especially in its late stages, has been associated with significant morbidity and mortality, often due to cardiovascular problems. Complications due to cardiovascular events are considered quite significant in children with CKD because they possess a higher rate of mortality compared to the mortality caused by CKD itself [1,2].* (Page 3, line 40) 🡪 **previously only supported by reference number one from Shulman et al. (1989)**

*The elevated mortality risk is largely attributed to factors such as accelerated atherosclerosis, vascular calcification, and chronic inflammation, which are common in CKD patients. Among these, cardiovascular disease stands out as a major in individuals with end-stage CKD. Both accelerated atherosclerosis and cardiovascular disease are major causes of morbidity and mortality in patients with end-stage CKD [3,4].* (Page 3, line 49) 🡪 **previously only supported by reference number three from Kumar et al. (2009)**

*CIMT reflects arterial wall thickness, making it a reliable diagnostic tool [8,9].* (Page 4, line 60) 🡪 **previously only supported by reference number eight from Chen et al. (1999)**

***New added articles within the references list:***

*2.	Li L-C, Tain Y-L, Kuo H-C, Hsu C-N. Cardiovascular diseases morbidity and mortality among children, adolescents and young adults with dialysis therapy. Front Public Health. 2023;11: 1142414. doi:10.3389/fpubh.2023.1142414*

*4.	Marx-Schütt K, Cherney DZI, Jankowski J, Matsushita K, Nardone M, Marx N. Cardiovascular disease in chronic kidney disease. European Heart Journal. 2025; ehaf167. doi:10.1093/eurheartj/ehaf167*

*9.	Akinmolayan A, Papacosta AO, Lennon LT, Ellins EA, Halcox JPJ, Whincup PH, et al. Carotid Intima‐Media Thickness, Carotid Distensibility, and Incident Heart Failure in Older Men: The British Regional Heart Study. JAHA. 2025; e037167. doi:10.1161/JAHA.124.037167*

*21.	Hussain Z, Bajeer I, Khatri S, Mohsin S, Kumar P, Hashmi S. Carotid Intima-Media Thickness in Children with Chronic Kidney Disease as an Early Predictor of Cardiovascular Diseases. PJMHS. 2023;17: 65–68. doi:10.53350/pjmhs02023171265*

*22.	Rizikalo A, Coric S, Matetic A, Vasilj M, Tocilj Z, Bozic J. Association of Glomerular Filtration Rate and Carotid Intima-Media Thickness in Non-Diabetic Chronic Kidney Disease Patients over a 4-Year Follow-Up. Life. 2021;11: 204. doi:10.3390/life11030204*

*23.	Palupi-Baroto R, Hermawan K, Murni IK, Nurlita T, Prihastuti Y, Puspitawati I, et al. Carotid intima-media thickness, fibroblast growth factor 23, and mineral bone disorder in children with chronic kidney disease. BMC Nephrol. 2024;25: 369. doi:10.1186/s12882-024-03771-z*

*24.	Colbert G, Jain N, De Lemos JA, Hedayati SS. Utility of Traditional Circulating and Imaging-Based Cardiac Biomarkers in Patients with Predialysis CKD. Clinical Journal of the American Society of Nephrology. 2015;10: 515–529. doi:10.2215/CJN.03600414*

(Page 17 – 18, line 281 – 361)
